# Monocyte Involvement in the Pathogenesis of Myeloproliferative Neoplasms

**DOI:** 10.3390/ijms26136422

**Published:** 2025-07-03

**Authors:** Xuedong Li, Mingli Xu, Yingying Wang

**Affiliations:** 1 Department of Immunology, School of Basic Medical Sciences, Chongqing Medical University, Chongqing 400016, China; 2 Chongqing Key Laboratory of Basic and Translational Research of Tumor Immunology, Chongqing Medical University, Chongqing 400016, China

**Keywords:** myeloproliferative neoplasms, monocytes, SLAMF7, Tie2

## Abstract

Classical BCR-ABL-negative myeloproliferative neoplasms are a heterogeneous group of hematologic malignancies, including essential thrombocythemia, polycythemia vera, and primary myelofibrosis. Monocytes, immune cells derived from hematopoietic stem cells, exhibit significant heterogeneity and contribute to immune regulation through cytokine secretion and differentiation into dendritic cells and macrophages. Aberrant monocytes are associated with the prognosis of MPNs, particularly PMF. Furthermore, these altered monocytes play a critical role in the pathogenesis and progression of MPNs. This review aims to explore the heterogeneity of different monocyte subsets during homeostasis and focuses on the potential mechanisms by which monocytes contribute to the development and progression of MPNs.

## 1. Introduction

Myeloproliferative neoplasms (MPNs) are a major subgroup of chronic myeloid neoplasms, alongside myelodysplastic syndromes (MDS), MDS/MPN overlap syndromes, and myeloid/lymphoid neoplasms, with eosinophilia associated with recurrent *PDGFRA*, *PDGFRB*, *FGFR1*, or *PCM1-JAK2* rearrangements. MPNs encompass seven clinicopathologic entities: chronic myeloid leukemia (CML), chronic neutrophilic leukemia (CNL), chronic eosinophilic leukemia not otherwise specified (CEL-NOS), myeloproliferative neoplasm unclassifiable (MPN-U), polycythemia vera (PV), essential thrombocythemia (ET), and primary myelofibrosis (PMF), all of which are classified as classical BCR-ABL negative MPNs [1,2]. This review focuses on these classical MPNs, which are driven by mutations in hematopoietic stem cells (HSCs), primarily involving Janus kinase 2 (*JAK2*), calreticulin (*CALR*), and myeloproliferative leukemia virus (*MPL*) gene [3]. *JAK2V617F* is the most prevalent mutation, detected in over 95% of PV patients and approximately 50% of ET and PMF cases [4]. Non-driver mutations, including *ASXL1*, *EZH2*, *TET2*, and *U2AF1*, further contribute to disease progression [5]. Clinically, patients present with constitutional symptoms such as fatigue, fever, night sweats, weight loss, pruritus, and early satiety, as well as complications including thrombosis and bleeding. Symptom severity correlates with tumor burden and comorbidities, with disease progression potentially leading to bone marrow failure or transformation to acute leukemia, significantly increasing mortality risk [6,7].

MPNs are also regarded as an inflammatory tumor disease. Patients with MPN often exhibit elevated levels of pro-inflammatory cytokines, chemokines, and growth factors such as IL-1β, IL-6, IL-8, IL-10, TNF-α, and TGF-β in the bone marrow microenvironment and peripheral blood [8,9,10]. This pro-inflammatory profile exacerbates genetic and epigenetic instability in MPNs, leading to cardiovascular events, fibrosis progression, and leukemic transformation in patients [11]. Additionally, various immune cells are dysregulated in MPNs, including neutrophils, lymphocytes, regulatory T cells, dendritic cells (DC), monocytes and myeloid-derived suppressor cells (MDSC) [12]. One recent study of bone marrow mononuclear cells in PMF patients with single cell sequence technology identified 15 subsets of T and NK cells and eight CD14+ monocyte subsets. Functional module analysis demonstrated an increased cytotoxic score of most T cells and a higher prevalence of LAG3 expressing effector T cells in overt-PMF compared to pre-PMF. An increased frequency of Mono 3 was also noticed in overt-PMF that expressed M-MDSC markers [13]. Several studies focusing on monocytes have demonstrated their abnormality, both in number and quality, which correlates strongly with MPN prognosis, particularly PMF prognosis [14,15]. Monocytes are unique due to their high plasticity in differentiating into fibrocytes, osteoclasts, DC, or macrophages in MPNs [16,17]. Furthermore, they perform essential functions, such as antigen presentation, migration, cytokine secretion, and interaction with T cells [18,19]. Several studies focusing on monocytes have revealed that abnormal monocytes may play a significant role in MPN progression through pro-angiogenesis by expressing Tie2 [20,21], pro-fibrosis by expressing SLAMF7 [22], pro-tumor activity via cytokine secretion such as TNF-α [23], and inhibiting T cell function through increased expression of PDL1 [24,25], as well as modulating the tumor microenvironment by differentiating into tumor-associated macrophages, etc. [26]. Therefore, this review aims to summarize recent advances in the biology of monocytes and particularly their involvement in pathophysiology of MPNs. The plasticity and heterogeneity of monocytes contribute to their highly complex functions in different tissues under various stimuli. Initial studies have demonstrated the phenotypic and transcriptional disturbances of monocytes in MPNs; however, the origins of these disturbances and their intricate roles in the pathogenesis and progression of MPNs still need to be elucidated. A summary of the advancements in understanding the underlying mechanisms could help guide further studies aimed at unmasking these complex processes, offering new therapeutic targets and proposing innovative treatment approaches in the future.

## 2. Overview of Monocytes

### 2.1. Monocyte Development and Differentiation

Distinct developmental pathways of monocytes were proposed using mouse models with novel experimental approaches such as fate mapping and single cell analysis [20]. Monocytes are derived in the bone marrow from HSCs, which differentiate into multipotent progenitor cells (MPPs) and then further into common myeloid progenitor cells (CMPs). These cells subsequently differentiate into granulocyte–macrophage progenitors (GMPs) or monocyte–dendritic cell progenitors (MDPs). MDPs could differentiate directly into Ly6cHi monocytes; however, GMPs eventually become monocyte progenitors (cMOPs) and ultimately mature monocytes [27,28]. Circulating monocytes can migrate into tissues in response to various signals, replenishing tissue macrophages or differentiating into monocyte-derived dendritic cells (mo-DCs) to transport antigens to lymph nodes or present them to T cells. Monocytes play a crucial role in the immune response by recognizing danger signals through pathogen pattern receptors. They can phagocytose, present antigens, secrete cytokines, and migrate into tissues [29].

### 2.2. Monocyte Heterogeneity and Its Homeostasis

Currently, there are three main subsets of human monocytes: classical monocytes (CMs: CD14++CD16−), intermediate monocytes (ICMs: CD14+CD16+), and non-classical monocytes (NCMs: CD14+CD16++) [29]. In healthy individuals, CMs typically account for approximately 85% of the monocyte population, while ICMs and NCMs comprise around 5% and 5–10%, respectively [30]. CMs have a relatively short lifespan in the bloodstream, surviving for about 1.6 days, after which the majority exit circulation to migrate into tissues. In contrast, circulating ICMs and NCMs last approximately 4 days and 7 days, respectively [31]. Notably, there is a linear relationship between these subsets, with CMs transitioning into ICMs, and subsequently into NCMs [32].

The transition from CMs to ICMs and NCMs occurs primarily in the diaphysis, as evidenced by Bianchini et al., who evaluated the distribution of monocyte subsets. They found the proportions of CMs, ICMs, and NCMs in the diaphysis to be 14:1:3, compared to 2:1:2 in the epiphysis. Further research indicated that the number of transitional zone (TZ) vessels within the bone marrow correlates positively with the total amount of PD-L1+ cells, which include both ICMs and NCMs, with NCMs predominating. Circulating CMs preferentially localize to the diaphyseal marrow, remaining in this region for extended periods before transiting into ICMs and NCMs following interactions with TZ vessels [33].

Monocytes maintain a homeostatic state, which can be disrupted under various conditions, such as chronic inflammation or infection, a topic that has been extensively reviewed elsewhere [34,35].

### 2.3. Phenotypic Features of Monocytes

Human peripheral blood monocytes express a variety of surface markers, including cluster differentiation antigens, chemokine receptors, and cytokine receptors [18]. Key markers include CD14, CD16, CD64 (Fc gamma RI), CD192 (also known as CCR2, a crucial mediator of monocyte migration), CX3CR1 (the fractalkine receptor), HLA-DR (which is expressed at the highest levels in the intermediate monocyte population), and CD195 (CCR5). Additionally, monocytes express TNFR1 (CD120a) and TNFR2 (CD120b) [36]. Recent studies on the immunophenotype of peripheral blood monocytes have shown that different subsets exhibit distinct phenotypic characteristics [30,31]. CMs particularly exhibit high level expression of CD11b, CD15s, and SSEA-1, CD35 (complement receptor type 1), CD36, CD38, CD49e (integrin α-5), CD89 (immunoglobulin-α Fc receptor), CDw93, CD96, CD99, CD114, CD182 (CXCR2), CD166 (ALCAM), CD181 (CXCR1), and leukotriene B4 receptor 1 (BLTR1), which also significantly differentiates them from the intermediate subset. ICMs express relatively high levels of CD32, CD39 (NTPDase-1), CD54, CD72, CD74, CD91 (α2-macroglobulin receptor), CD105 (endoglin), CD163, CD195 (CCR5), CD275, CD305 (LAIR1), CDw328 (Siglec-7), and HLA-DR/DQ. NCMs present the high expression levels of CD29 (integrinβ-1), CD31 (PECAM1), CD45RA, CD75, CD120b (TNFR2), CD132 (interleukin IL-2 receptor subunit gamma), CD244 (SLAMF4), CD294, and HPC (hematopoietic progenitor cell) [37,38].

### 2.4. Functional Features of Monocytes

#### 2.4.1. Cytokine Secretion

Cytokine production in response to Toll-like receptor (TLR) agonists is a prominent feature of monocytes and macrophages. Under stimulation with TLR1-9 agonists, CMs secrete significantly higher levels of IL-1β, IL-6, and TNF-α compared to NCMs and ICMs, with NCMs exhibiting the weakest secretion ability [28,39]. Notably, when stimulated with TLR4 agonists, CMs can produce large amounts of TNF-α, IL-6, and IL-1β [39]. Studies have shown that, upon exposure to increasing concentrations of lipopolysaccharide (LPS), CMs generate the highest levels of G-CSF, IL-10, CCL2, and IL-6 compared to ICMs and NCMs. Conversely, NCMs are more adept at producing inflammatory cytokines like TNF-αand IL-1, while ICMs have the least capacity for cytokine secretion [39]. Additionally, LPS-stimulated DCs derived from CD16− monocytes produce higher levels of IL-12 mRNA and lower levels of TGF-β1 mRNA compared to DCs derived from CD16+ monocytes, with corresponding protein levels confirmed [40].

#### 2.4.2. Differentiation Potential

Monocytes can differentiate into monocyte derived dendritic cells (mo-DCs) and macrophages under various culture conditions, but their differentiation potential varies among the three subsets. Comparative studies indicate that CMs fully differentiate into mo-DCs after 1 week, while only a limited number of NCMs and ICMs undergo this differentiation, with most cells undergoing apoptosis [39]. Functional assays have demonstrated that these differentiated cells exhibit defects in inducing T-cell proliferation or IFN-γ production. None of the three monocyte subsets can differentiate into plasmacytoid dendritic cells (p-DCs) [39]. Furthermore, mo-DCs derived from CD16+ monocyte express higher levels of CD86, CD11a, and CD11c, while showing lower expression of CD1a and CD32, compared to mo-DCs derived from CD16− monocytes. When co-cultured with T cells, both mo-DCs derived from CD16+ and CD16− monocytes induce T-cell proliferation without significant differences; however, mo-DCs derived from CD16+ monocytes promote higher secretion of IL-4 and IFN-γ from T cells [40].

Regarding differentiation into macrophages, all monocyte subsets adopt macrophage morphology, characterized by a rounded, firmly adhered appearance with actin-rich podosomes and extensions for substrate adhesion. Compared to M1 and M2 macrophages generated from ICMs and NCMs, M1 and M2 macrophages derived from CMs exhibit stronger phagocytic abilities and secrete higher levels of cytokines such as IL-10, IL-6, and PDGF-BB [39]. Under homeostatic conditions, classical monocytes can maintain their monocyte-like state without differentiating into macrophages or DCs, acting as a local reservoir and participating in non-immune tissue surveillance by transferring antigen information to lymph nodes [41].

#### 2.4.3. Antigen Presentation

In vitro studies have evaluated the ability of mo-DCs derived from CD16− and CD16+ monocytes to prime autologous and allogeneic CD4 T cells, revealing that mo-DCs from CD16+ monocytes elicit a relatively higher autologous response and a similar allogeneic response compared to mo-DCs derived from CD16− monocytes. mo-DCs derived from CD16+ monocytes consistently induce higher secretion of IL-4 by T lymphocytes, while the levels of IFN-γ produced by both mo-DC types are comparable [40]. Beyond their role as antigen-presenting cells, monocytes themselves exhibit significant antigen-presenting capabilities. Animal studies suggest that monocytes can capture and present antigens in vivo, functioning similarly to conventional DCs [42]. Other studies indicate that monocytes primarily transport antigens to lymph nodes, where they present them to other antigen-presenting cells, such as conventional dendritic cells (cDCs) [42,43,44]. However, whether human monocytes in vitro could present antigen to T cells is still unknown, and needs to be further investigated.

#### 2.4.4. Migration and Extravasation

After being produced in the bone marrow, monocytes enter peripheral blood circulation and rapidly migrate to damaged tissues in response to inflammatory or infection signals, playing a crucial role in immune defense. Mouse model studies demonstrated that monocyte migration is primarily regulated by specific chemokines. For instance, when CCR2 on monocytes binds to MCP-1, it triggers the release of monocytes from the bone marrow [45]. Similarly, CCL19 binds to CCR7 on monocyte surfaces to guide them to lymph nodes, where they perform their functions. Upon reaching target sites, monocytes can differentiate according to the local microenvironment, such as becoming macrophages that phagocytose pathogens, clear cellular debris, and secrete anti-inflammatory cytokines [46]. The migration ability of human monocytes was evaluated using transwell and transendothelial migration assays. The results revealed that CD16− and CD16+ monocytes are guided by different chemokines. CD16- monocytes preferentially respond to MCP-1 and MIP-1, while CD16+ monocytes, which express high levels of CX3CR1, are guided by the CX3CR1-FKN axis [47]. Other chemokines, such as CCR5 and CCR6, also play roles in the migration and extravasation of monocytes [46]. Further studies focusing on the migration of human monocytes are needed.

### 2.5. Transcriptional Features of Monocytes

Numerous studies have demonstrated transcriptional differences among human monocyte subsets [48,49,50,51,52]. CMs primarily participate in inflammatory responses and tissue repair, exhibiting gene expression profiles associated with these functions. Notably, CMs highly express genes from the S100 protein family, such as S100A8, and S100A12, which encode proteins crucial for signal transduction and cell recruitment during inflammation [49,50,51]. Additionally, they express angiogenesis and tissue repair-related genes, including *VEGF*, and *TGF-β*, which facilitate the repair and reconstruction of damaged tissues [50]. CMs also display elevated expression of neuregulin 1 (*NRG1*), phospholipase A2 group VII (*PLA2G7*), *ADAM19*, low-density lipoprotein receptor (*LDLR*), scavenger receptor class B member 1 (*SCARB1*), and stabilin 1 (*STAB1*), all of which mediate inflammatory responses and participate in immune regulation [49].

ICMs play a vital role in antigen presentation, characterized by their highest expression of MHC-II among monocyte subsets, which enables them to effectively present antigens to T cells [48,52]. Furthermore, ICMs present the highest expression of genes involved in cell differentiation and cell function such as *IRF5*, *IRF8* and *NFKB1* [52].

In contrast, NCMs exhibit high expression of cytoskeletal regulatory genes, including CDC42 effector protein-4 (*CDC42EP4*), creatine kinase B (*CKB*), EMAP-like protein 4 (*EML4*), Enah/vasodilator-stimulated phosphoprotein-like (*EVL*), formin-like 2 (*FMNL2*), metastasis suppressor-1 (*MTSS1*), and *RHOC* [50]. These genes are involved in the migration and adhesion of non-monocytes, enabling NCMs to function as efficient patrolling cells that can rapidly migrate to sites of infection. Additionally, NCMs express genes related to both inflammatory and anti-inflammatory states, such as heme oxygenase-1 (*HMOX1*), *KLF1*, and *FCGR3B*, reflecting their capability to modulate immune responses according to the tissue environment [49,51].

## 3. Monocyte Characteristics in MPNs

A significant decrease in CMs and an increased frequency of ICMs and NCMs was noticed in PMF patients after analysis of peripheral blood monocytes using flow cytometry. Further feature analysis of these three monocyte subsets using CCR2, CXC3R1, CCR5, TNFR1, and TNFR2 demonstrated that there was an increased proportion of CCR2+, and CCR5+ monocytes among CMs, ICMs, and NCMs, while the frequency of CX3CR1+ monocytes in NCMs cells declined. Decreased frequency of TNFR1+ monocytes and increased frequency of TNFR2+ monocyte was observed in PMF patients before treatment, compared to healthy donors [53]. A study conducted in Brazil analyzed the phenotypic features of peripheral monocytes in patients with MPNs compared to healthy donors using specific markers: CD14, CD16, CD64, CD80/86, HLA-DR, and CD56. The results showed an increased frequency of ICMs and NCMs, along with a decreased frequency of CMs in PV, ET, and PMF. Additionally, the frequency of ICMs correlated with the presence of the JAK2 mutation. The study also found an increased frequency of CD56+ aberrant monocytes, while patients with PV and ET exhibited a reduced frequency of CD80/86+ monocytes. However, there was no significant difference in the frequency of CD64+ monocytes between the groups [15]. Moreover, PMF monocytes showed impaired differentiation into dendritic cells (DCs), primarily characterized by the presence of immature DCs that continued to express CD14, while CD80 expression was reduced. In mature DCs, the expression of both CD40 and CD80 was also decreased [53]. Collectively, peripheral blood monocytes from MPN patients exhibited a significant decrease in CMs and an increase in ICMs and NCMs, along with altered expression of various surface markers, including elevated levels of CCR2 and CCR5, reduced TNFR1, increased TNFR2, and impaired differentiation into dendritic cells, particularly in patients with PMF.

Transcriptomic sequencing of CD163+ monocytes isolated from the bone marrow of MPN patients revealed that, in PV, the upregulated pathways included cytokine–cytokine receptor interactions, cytokine receptor pathways, the Rap1 signaling pathway, the cGMP-PKG signaling pathway, platelet activation, and complement and coagulation cascades. The downregulated pathways were associated with ribosomal function, the cell cycle, oxidative phosphorylation, and pathways related to neurodegeneration. In ET, the upregulated pathways included platelet activation, cell adhesion molecules, ECM-receptor interactions, focal adhesion, and regulation of the actin cytoskeleton. The downregulated pathways involved viral protein interactions with cytokines and their receptors, oxidative phosphorylation, thermogenesis, as well as the NF-kappa B signaling pathway, TNF signaling pathway, and IL-17 signaling pathway. In PMF, the upregulated pathways related to cytokine–cytokine receptor interactions, platelet activation, the JAK-STAT signaling pathway, complement and coagulation cascades, and Fc gamma R-mediated phagocytosis. In contrast, the only downregulated pathways were associated with coronavirus disease (COVID-19) and ribosomal function [54].

In another study, CD14+ monocytes were isolated using magnetic bead sorting and compared with healthy monocytes. A total of 101 differentially expressed genes (DEGs) shared among ET, PV, and PMF were primarily enriched in pathways related to coagulation, complement activation, JAK-STAT signaling, and TNF/NF-kB signaling. In PMF patients, the upregulated genes in monocytes were mainly associated with platelet function, including *PPBP*, *ITGB3*, and *PF4*, while the downregulated genes primarily related to lymphoid and erythroid lineages, such as *DNTT*, *VPREB*, various immunoglobulin transcripts, and *GYPA* [55]. Additionally, a bioinformatics analysis of peripheral blood monocytes from MPN patients in which the top 20% of differentially expressed genes underwent enrichment analysis revealed significant associations with KRAS signaling, immune activation, response to hypoxia, stimuli of degranulation, complement system regulation, heme metabolism, and regulation of cell death. Subgroup analyses demonstrated that PV patients exhibited greater enrichment in the IFN-γ pathway, ET patients were more associated with IL-2-STAT5 signaling and fatty acid metabolism, and PMF patients displayed enhanced KRAS signaling, heme metabolism, and stimulation of neutrophil degranulation [56].

## 4. Mechanism of Monocyte Involvement in MPN Progression

As described above, monocytes have been implicated in the prognosis of MPNs, particularly in PMF. In MPNs, monocytes demonstrate a redistribution of monocyte subsets, dysfunction of mo-DCs, and transcriptional dysregulation. This shift in monocyte populations is associated with aberrant functions that may contribute to MPN pathogenesis, although the underlying mechanisms remain to be fully elucidated. Several initial studies demonstrated that monocytes play a multifaceted role in the progression of MPNs through various mechanisms, as described in Table 1:

- Pro-Angiogenic Activity: Monocytes may exhibit pro-angiogenic properties by expressing Tie2 or even differentiating into endothelial cells, thereby promoting vascularization and tumor progression [20,21].
- Pro-Fibrotic Effects: Through the expression of SLAMF7 and differentiation into fibrocytes, monocytes may contribute to fibrotic processes within the bone marrow microenvironment [22].
- Pro-Tumoral Effects: Monocytes can express programmed death-ligand 1 (PD-L1), which directly inhibits T cell function, thereby facilitating tumor progression. This immune evasion mechanism allows tumors to escape immune surveillance [24,25].
- Cytokine Secretion: An imbalance in monocyte subsets can lead to abnormal cytokine secretion, particularly an excess of TNF-α. This cytokine is known to promote MPN progression. Additionally, intrinsic disturbances in monocyte function may result in abnormal interactions with T cells, further contributing to tumor growth [23].
- Differentiation Potential: Monocytes present the ability to differentiate into various cell types, including DCs, macrophages. These differentiated cells can interact with T cells, potentially inhibiting their functions and facilitating tumor evasion [55].
- Osteoclast Differentiation: Monocytes can also differentiate into osteoclasts, which may play a significant role in the progression of MPNs by affecting the bone marrow microenvironment [57].
- Senescence and Dysfunction: Monocytes may enter a senescent state, leading to mitochondrial dysfunction. This impairment can result in abnormal migration, antigen presentation, and overall immune response, further complicating their role in MPNs [58,59].

**Figure 1:**
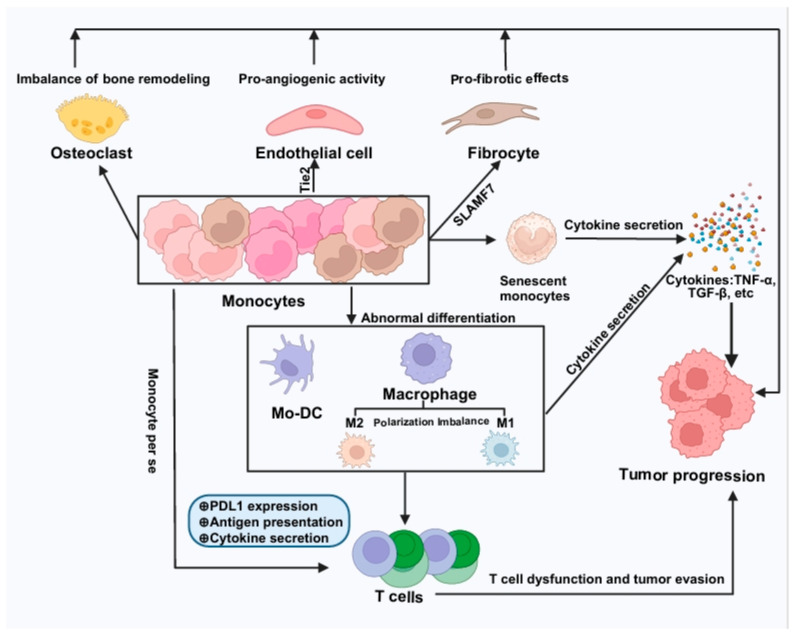
Mechanisms of monocyte involvement in MPN pathophysiology. Monocytes play diverse roles in the pathogenesis of myeloproliferative neoplasms (MPN). Certain monocytes express Tie2 or SLAMF7, exhibiting pro-angiogenic and pro-fibrotic activities that promote tumor growth. Additionally, monocytes can differentiate into osteoclasts, impacting the bone marrow microenvironment and further advancing MPNs. Monocytes also express PD-L1 and exhibit aberrant antigen presentation, directly suppressing T cell activity and facilitating immune evasion. Furthermore, monocytes can differentiate into mo-DCs and macrophages, which inhibit T cell functions and promote immune evasion through co-inhibitory molecules, defective antigen presentation, and abnormal cytokine secretion. Unhealthy monocytes, including senescent forms, secrete high levels of cytokines such as TNF-α, TGF-β, IL-6, and IL-1β, contributing to MPN progression. Collectively, these mechanisms underscore the complex roles of monocytes in the pathogenesis of MPNs.

These potential mechanisms underscore the complex contributions of monocytes to MPN progression and highlight the necessity for further research to fully understand their roles in disease pathology. A summary of these underlying mechanisms is illustrated in Figure 1.

### 4.1. Pro-Angiogenesis via Expressing Tie2

The Tie2 receptor, a receptor tyrosine kinase, plays a crucial role in regulating physiological processes such as vascular growth and differentiation in recognizing their ligands such as angiopoietin-2. Its activation triggers a cascade of downstream signaling pathways that are essential for angiogenesis, the formation of new blood vessels, which is a critical rate-limiting step in tumor development. Tie2 is frequently expressed on vascular endothelial cells and some hematopoietic stem cells, particularly monocytes or macrophages [60]. These Tie2+ cells account for 35% to 75% of the CD14lowCD16+ monocytes, so this subset of monocytes expressing Tie2 is known as Tie2-expressing monocytes (TEMs) [61]. Tie2+ cells account for 1.6% to 7.4% of the total PBMCs, and further evaluation with PBMCs from cancer patients demonstrated that the frequency of TEMs ranged from 1.8% to 10.1% of the total PBMCs. Further studies using tumor mouse models have demonstrated that the selective elimination of TEMs effectively reduces tumor angiogenesis, leading to tumor regression [62]. Initial clinical studies with the Tie2 kinase inhibitor rebastinib in treating myeloid malignancies and breast cancer demonstrated good tolerance, but its efficacy needs further evaluation with large sample sizes [63,64]. However, the existence and functional significance of Tie2+ macrophage in tumor environment is still the subject of debate [65], and therefore requires further study.

In relation to involvement in MPNs, there are only two clinical observations. An increased frequency of ICMs expressing Tie2 has been noted in the peripheral blood of patients with PMF [20]. Additionally, their frequency was found to be higher in spleen tissue-derived mononuclear cells (MNCs) from PMF patients compared to those of controls [21]. These preliminary findings suggest that Tie2+ monocytes may promote the progression of MPNs through their angiogenic properties, potentially contributing to complications such as thrombosis and splenomegaly.

### 4.2. Pro-Fibrosis via Expressing SLAMF7

SLAMF7, a member of the SLAM family, primarily induces B cell proliferation and autocrine signaling. Additionally, it plays a negative regulatory role in T-cell responses, inhibiting their activity and contributing to the modulation of pro-inflammatory responses in monocytes [66]. SLAMF7 is expressed on various immune cells, including plasma cells, NK cells, plasmacytoid dendritic cells (pDCs), T cells, B cells, monocytes, and mo-DCs. It is also highly expressed in tumor cells, particularly in multiple myeloma, and on the surface of fibroblasts in both mice and humans [67]. In MPNs, monocytes have been found to express elevated levels of SLAMF7, and an increased frequency of CD14+SLAMF7+ monocyte was noticed in MPN patients including PV and ET patients [22,68]. Specifically, SLAMF7highCD14+ monocytes are considered precursor cells for human fibrocytes, and their numbers significantly increase in MF patients, particularly in those with the JAK2V617F mutation [22]. Further research has demonstrated that the JAK2V617F mutation directly leads to increased SLAMF7 expression in monocytes [22]. The elevated presence of SLAMF7high monocytes with a higher JAK2V617F allelic burden is associated with the development of PMF. This suggests that monocytes contribute to MPN pathogenesis through their differentiation into fibrocytes, promoting bone marrow fibrosis. Thus, SLAMF7 serves as a potential mechanism by which monocytes facilitate the fibrotic processes observed in MPNs. Ultimately, antibody targeting of SLAM7, such as via elotuzumab, could be a candidate treatment for PMF, as elotuzumab has demonstrated its benefits in multiple myeloma [69]. Currently, there is one active clinical trial involving wlotuzumab for treating PMF, but it has not yet begun recruiting patients.

### 4.3. Protumoral Effect via Expressing PD-L1

PD-L1 plays a critical role in tumor immune evasion by binding to PD-1 on the surface of T cells, thereby inhibiting T cell-mediated immune responses against tumors [70]. Numerous studies have indicated that this effect is primarily attributable to PD-L1 expression on tumor cells. Additionally, several studies have emphasized that PD-L1 is also expressed on the surface of antigen-presenting cells, where it plays a crucial role in the effectiveness of immune checkpoint inhibitor therapies [71,72]. Research by M. Bianchini et al. has shown that PD-L1 is predominantly expressed on non-classical monocytes, with minimal or absent expression on intermediate and classical monocytes, particularly in the context of cardiovascular diseases [33]. Under inflammatory conditions, monocytes can be activated by various cytokines, leading to the upregulation of PD-L1 expression, which subsequently suppresses T cell immune responses. Notably, the PD-L1 gene is located on chromosome 9, adjacent to the JAK2 gene [73]. Studies have demonstrated a correlation between JAK2 mutations and PD-L1 expression in both melanoma and MPNs [25,74]. For instance, a study on melanoma indicated that mutations in JAK1/JAK2 may contribute to resistance against PD-1 antibody treatments [74]. In the context of MPNs, research has shown that JAK2 mutations are associated with increased PD-1 expression on the surface of various cell types, including monocytes, MDSCs, and megakaryocytes [75]. For MPNs associated with CALR mutations, studies suggest that in vivo blockade of PD-1 can help restore or enhance T cell responses [25]. Two initial clinical trials conducted in 2021 indicated that PD-1 inhibitors could establish a new standard of care for patients with MPNs, potentially improving survival rates and enhancing quality of life. Additionally, a Phase II study investigating the combination of fedratinib and nivolumab in patients with myelofibrosis began in 2022 and was expected to conclude in December 2024 [76,77]. However, these PD-1 inhibitors are not specifically targeting PD-L1 on monocytes; rather, they primarily act on PD-1 expressed on T cells. This may help explain their limited efficacy in MPNs. In the future, antibodies or inhibitors designed to target PD-L1 on monocytes could represent a promising therapeutic approach.

### 4.4. Tumorigenic Effects Associated with Aberrant Cytokine Secretion

Although various cytokine disturbances have been observed in MPN patients, particularly in TNF, IL-1β, IL-8, and IL-6, studies investigating the cytokine secretion capabilities of monocytes remain somewhat controversial. An initial study in 2017 using the ELISA method found that IL-8 levels in monocytes after in vitro culture from MF patients were elevated compared to those in healthy monocytes, both in LPS-stimulated and non-stimulated groups. Additionally, the TNF-α level increased after LPS stimulation compared to non-stimulated groups, However, these monocytes were cultured in vitro for 30 days, which likely did not accurately reflect the true characteristics of monocytes from patients with PMF [78]. In 2018, Fisher DAC et al. demonstrated that 14 out of 15 cytokines were overproduced in MF patients upon stimulation, with monocytes identified as the principal cellular source for most of these cytokines, as determined by mass cytometry. The most prominent cytokines included TNF-α, IL-8, and IL-1RA. Moreover, TNF-α could induce a limited set of cytokines in monocytes, such as IL-8/CXCL8, IL-6, CCL4/MIP-1β, and IL-1RA [79]. The elevated concentrations of TNF-α in MPN patients are attributed to a defect in the negative regulation of Toll-like receptor (TLR) signaling in monocytes, leading to insensitivity to IL-10 and resulting in unchecked TNF-α production upon TLR activation [80]. In a subsequent study in 2020, Barone M et al. found that intracellular cytokine staining revealed a decrease in the proportions of IL-1β+, TNF-α+, and IL-6+ monocytes in MPN patients compared to healthy individuals, both in LPS-stimulated and non-stimulated groups. Following treatment with ruxolitinib, these monocyte proportions showed some recovery. ELISA measurements indicated that, at baseline, the concentrations of IL-1β at 4 and 24 h, TNF-α at 4 h, IL-6 at 4 and 24 h, and IL-10 at 24 h were significantly reduced in the supernatants of MF monocytes compared to those of healthy controls. Notably, TNF-α levels at 24 h demonstrated a slight increase in MF monocytes compared to healthy donors [53]. Collectively, MF monocytes are likely to secrete elevated levels of TNF-α and IL-8 following prolonged or persistent TLR stimulation, which may contribute to the progression of MPNs and could be considered as a treatment target for these diseases [81,82,83,84]. For instance, IL-8, via its receptor CXCR2, facilitates the infiltration of inflammatory cells into the bone marrow, promoting the expansion of mutated HSCs and exacerbating fibrosis [83,84]. Furthermore, blocking the CXCR1/2 axis using reparixin has shown promise in reducing bone marrow fibrosis in mouse models. An ongoing phase II clinical trial evaluating reparixin in patients with PMF is expected to be completed in 2026, which could provide further insights into its therapeutic potential.

### 4.5. Over-Differentiation Towards Osteoclasts

In MPNs, monocytes can abnormally differentiate into osteoclasts, leading to an imbalance between osteoclasts and osteoblasts, which contribute to the development of bone marrow fibrosis [85]. Research indicates that JAK2+ monocytes exhibit a greater propensity to differentiate into osteoclasts compared to normal monocytes. This increased differentiation results in an enriched osteoclast environment that promotes the proliferation and survival of mutated cells associated with MPNs [57,85]. Interestingly, studies have shown that osteoclasts in the context of MPNs are functionally impaired, primarily exhibiting a reduced capacity for bone resorption. This impairment can lead to a shift in the balance of bone remodeling towards bone regeneration, allowing osteoblasts to prevail, and ultimately resulting in bone sclerosis [17]. However, the proliferation of osteoblasts can further promote the development of fibrosis, as excessive osteoblast activity perpetuates the proliferation of clonal MPN cells [57]. The abnormal differentiation of monocytes into osteoclasts significantly impacts the progression of MPNs by disrupting the bone marrow structure, promoting fibrosis, exacerbating osteoporosis, and altering the microenvironment [86]. The cytokines and signaling pathways involved in this process present potential targets for future therapeutic strategies. Modulating osteoclast function may emerge as an important approach to inhibit the progression of MPNs, offering new avenues for treatment and management of the disease.

### 4.6. Abnormal Differentiation Towards Macrophages

Monocyte-derived macrophages play a critical role in chronic inflammation and are highly heterogeneous, capable of rapidly polarizing into either M1 (pro-inflammatory) or M2 (anti-inflammatory) macrophages in response to microenvironmental signals. Tumor-associated macrophages (TAMs) have been shown to be significant in tumor immunology, and dysregulation in their differentiation and function is crucial in the advancement of MPNs [12]. Historical evidence from 1992 by Thiele et al. indicated that increased macrophage presence was observed in 25 out of 30 patients, suggesting their potential involvement in the generation of bone marrow fibrosis [87]. More recent studies by Molitor et al. reported a significant increase in CD68-positive macrophages in patients with PMF compared to those with chronic myeloid leukemia (CML) and healthy controls. Notably, the frequency of these macrophages in PMF was higher than in PV and ET, although CD68-positive macrophages were also elevated in PV compared to ET [88]. These macrophages can induce the proliferation of myofibroblasts via the vitamin D receptor, further contributing to fibrosis [89]. Transcriptomic analyses of isolated monocyte/macrophages from various MPN entities have highlighted their potential roles in MPN development [54]. In patients with ET, macrophages tend to polarize towards the M2 phenotype, which is generally associated with tissue repair, anti-inflammatory responses, and regulation of the tumor microenvironment. M2 macrophages inhibit excessive inflammatory responses by secreting anti-inflammatory cytokines such as IL-10 and TGF-β. Additionally, M2 macrophages are centrally involved in fibrosis through the secretion of pro-fibrotic cytokines in PMF, as well as in tissue regeneration and scar formation [54].

In experimental models, such as Asxl1^−/−^Jak2^V617F^ mice, the proportions of macrophages, predominantly M1 macrophages, were markedly increased in the bone marrow and spleen compared to Jak2^V617F^ mice. Further studies using a monocyte transfusion model demonstrated that the increased macrophages were neoplastic and derived from monocytes rather than primary tissue-resident macrophages [90]. These differentiated macrophages can further polarize into M1 macrophages under inflammatory stimulation, secreting cytokines that create a positive feedback loop, exacerbating the inflammatory response. However, further investigation is needed to determine whether there are differences in the differentiation capabilities of the three monocyte subpopulations in inflammatory environments and how the functions of the differentiated macrophages may change in the context of MPNs. Understanding these dynamics could provide insights into potential therapeutic targets for modulating macrophage activity in MPN patients.

### 4.7. Abnormal Differentiation Towards DCs

In vitro studies have shown that monocytes can differentiate into DCs with the assistance of granulocyte-macrophage colony-stimulating factor (GM-CSF) and interleukin-4 (IL-4). The IL-4 signal transmits through the JAK1/3 signaling pathway. However, in the presence of JAK2 mutations, monocytes exhibit increased sensitivity to GM-CSF, which leads to a reduced ability to differentiate into mo-DCs and a more pronounced macrophage-like phenotype [53]. In patients with MF, there is a notably decreased number of cDCs in peripheral blood compared to healthy donors (HD). Further phenotypic and functional assays of mo-DCs in vitro have demonstrated that monocytes from patients with the JAK2V617F mutation show defects in differentiating towards immature mo-DCs, characterized by a lack of upregulation of surface markers such as CD1a and CD80 [53]. Additionally, these monocytes exhibit decreased antigen presentation capabilities and increased endocytosis abilities, although their migratory capacity remains unaffected compared to HD. Interestingly, research indicates that ruxolitinib, the preferred treatment for MPNs, can adversely impact the differentiation and functionality of mo-DCs [91]. Mouse model experiments have further revealed that defects in DCs can lead to the development of myeloproliferative diseases (MPDs) by disrupting the interactions between DCs and CD4+ T cells [92]. Given these findings, targeting the differentiation of monocytes into mo-DCs and exploring the interactions between mo-DCs and CD4+ T cells may provide new insights and therapeutic strategies for treating MPNs. This approach could help address the dysregulated immune responses and inflammation associated with MPNs, potentially leading to more effective treatments.

### 4.8. Effect of Monocytes per Se

Monocytes are innate immune cells that play several critical roles beyond their differentiation into other cell types, such as DCs or macrophages. They possess the ability to perform endocytosis and present antigens without undergoing differentiation. Additionally, monocytes can contribute to thrombosis formation by secreting tissue factor (TF) and interacting with platelets. Their intrinsic state, including factors like senescence and metabolic disturbances, particularly abnormal mitochondrial function, can significantly influence the progression of MPNs.

Monocytes serve as a primary vascular source of TF [93]. In the context of MPNs, which are chronic inflammatory disorders, monocytes are continuously exposed to inflammatory stimuli, leading to the release of significant amounts of TF. When these monocytes migrate to sites of vascular endothelial injury, they facilitate the binding of TF with factor VIIa, activating thrombin and initiating the coagulation cascade. MPN patients often present with elevated platelet counts, particularly in ET. Platelets expressing P-selectin interact with monocytes, enhancing platelet activation and aggregation, which can lead to thrombosis, a common complication in patients with MPNs. Simultaneously, platelets can stimulate monocyte activation and promote its secretion of pro-inflammatory cytokines [94].

The healthy state of monocytes is crucial; senescent monocytes tend to secrete excess cytokines, such as TNF-α [95]. Mitochondria are the primary source of cellular energy, and mitochondrial dysfunction can significantly impact monocyte function. In monocytes, the transition from an inflammatory phenotype to an immunosuppressive state involves mitochondrial metabolic reprogramming. During inflammation, damaged mitochondria accumulate, leading to increased production of reactive oxygen species (ROS) and activation of inflammasome signaling [96]. This metabolic shift results in a transition from oxidative phosphorylation to glycolysis and lactate production, with mitochondrial ATP production being replaced by succinate oxidation, further stimulating ROS and inflammatory cytokine production. Elevated oxidative stress and mitochondrial dysfunction can lead to increased apoptosis of monocytes [97]. Such abnormalities in mitochondrial function contribute to the progression of chronic inflammatory diseases, including atherosclerosis and chronic kidney disease [98]. Given that MPNs can affect individuals of all ages, with a majority of patients being 60 years of age or older [99], the presence of more senescent monocytes with dysfunctional mitochondria is particularly concerning. Therefore, studying the impact of abnormal mitochondrial function in monocytes on the development of MPNs holds significant clinical and research value.

### 4.9. Potential Treatments Targeting Monocytes for MPNs

As described above, dysregulated monocytes may be involved in the pathophysiology of MPNs through several mechanisms, which could potentially serve as targets. These can be summarized into three major strategies: ① Elimination or Decrease of Pathogenic Monocytes: This can be achieved using inhibitors such as rebastinib, which targets Tie2, or monoclonal antibodies like elotuzumab, which targets SLAMF7. ② Neutralization of Adverse Effects Induced by Cytokines: This strategy involves targeting cytokines secreted by monocytes by utilizing inhibitors that affect IL-1β, CXCL8/CXCR, and TNF/TNFR-mediated pathways. ③ Reprogramming Dysregulated Monocytes: This strategy aims to convert dysregulated monocytes into a healthy state using small molecules, although this approach has not yet been extensively explored. However, there are only limited number of clinical studies targeting monocytes for MPN treatment. There remain numerous potential targets to explore for managing MPNs, alongside a deeper understanding of the role of monocytes in the pathophysiology of this disease.

## 5. Conclusions

Abnormalities in both the quantity and quality of monocytes are closely associated with the prognosis of MPNs. Several studies have investigated the mechanisms by which monocytes contribute to MPN progression. Aberrant monocytes are linked to disease advancement through both direct effects, such as pro-angiogenic activity via Tie2 expression, pro-fibrotic effects through SLAM7 expression, and tumorigenic processes driven by excessive cytokine production, as well as indirect effects, including PD-L1 expression that inhibits T cell function, and differentiation into osteoclasts, macrophages, and mo-DCs, all of which facilitate tumor evasion from immune surveillance. Moreover, the impaired functionality of monocytes, exacerbated by age-related abnormalities in mitochondrial quantity and quality, may significantly contribute to the initiation of MPNs. However, many questions remain to be elucidated. There may be differences between peripheral blood and bone marrow monocytes, and the presence of a small number of CD34+CD14+ cells within CD14+ monocytes complicates the analysis of true monocyte characteristics. Additionally, distinctions have not been made between genetically mutated clonal monocytes and relatively normal monocytes lacking gene mutations. The mechanisms underlying the production of abnormal monocytes in MPNs and their specific roles in the onset and progression of MPNs are still not fully understood, particularly in relation to whether monocytes in the bone marrow microenvironment are recruited or resident after differentiation from myeloid stem cells in response to local tumor microenvironmental cues. Therefore, it is crucial to comprehensively investigate the characteristics and underlying mechanisms of peripheral blood and bone marrow monocytes, especially their roles in the pathogenesis of MPNs. Addressing these questions will not only enhance our understanding of monocyte involvement in MPN development but also provide valuable insights for the development of novel therapeutic strategies. Additionally, this could enhance the understanding of their implications in other chronic myeloid neoplasms, such as CMML, MDS.

## Figures and Tables

**Table 1:** Summary of studies focusing on monocyte involvement in MPNs.

Monocyte Disturbance		Human Monocytes	Mouse Monocytes
Pro-angiogenesis (Tie2)		20, 21	
Pro-fibrosis (SLAMF7)		22, 67	22
Abnormal cytokine secretion (TNF-α, IL-1β, CXCL8)		53, 79, 80	
Inhibiting T cell function (PDL1)		73, 75	73
Abnormal differentiation	mo-DC	41	92
	Macrophages	42, 90	90
	Osteoclast	17, 57	
